# SARS-CoV-2 and Brain Health: New Challenges in the Era of the Pandemic

**DOI:** 10.3390/microorganisms11102511

**Published:** 2023-10-08

**Authors:** Waqas Ahmed, Jia Feng, Yifan Zhang, Lukui Chen

**Affiliations:** 1 Department of Neurosurgery, Neuroscience Center, Integrated Hospital of Traditional Chinese Medicine, Southern Medical University, Guangzhou 510310, China; 2 School of Medicine, Southeast University, Nanjing 210009, China; 3 Guangdong Second Provincial General Hospital, Guangzhou 510317, China

**Keywords:** SARS-CoV-2, COVID-19, CNS, BBB, transmission routes

## Abstract

Respiratory viral infections have been found to have a negative impact on neurological functions, potentially leading to significant neurological impairment. The SARS-CoV-2 virus has precipitated a worldwide pandemic, posing a substantial threat to human lives. Growing evidence suggests that SARS-CoV-2 may severely affect the CNS and respiratory system. The current prevalence of clinical neurological issues associated with SARS-CoV-2 has raised significant concerns. However, there needs to be a more comprehensive understanding of the specific pathways by which SARS-CoV-2 enters the nervous system. Based on the available evidence, this review focuses on the clinical neurological manifestations of SARS-CoV-2 and the possible mechanisms by which SARS-CoV-2 invades the brain.

## 1. Introduction

Respiratory diseases resulting from viral agents pose a significant challenge to public health. Such conditions annually contribute to elevated levels of illness and death, particularly among vulnerable populations such as youngsters, older adults, and individuals with compromised immune systems [1]. The primary viral agents responsible for respiratory diseases include coronavirus (CoV), influenza virus (IV), human respiratory syncytial virus (hRSV, orthopneumoviruses), and human metapneumovirus (hMPV, metapneumoviruses) [2]. The primary transmission mode for these viruses is through direct contact with contaminated objects or inhalation of suspended droplets [3,4].

All of these viruses share the common characteristic of being able to cause bronchiolitis and pneumonia, resulting in a significant number of hospitalizations during each winter season [5,6]. In addition to the seasonal viruses that affect the respiratory system, new strains of these viruses periodically spread among people, resulting in epidemics or even pandemics. Typically, these viruses belong to the RNA viral group, including influenza A subtypes and strains of human coronaviruses. These viruses exist within an animal reservoir and can traverse the species barrier, adapting to and infecting a new host [2,7]. The most recent emerging virus, known as severe acute respiratory syndrome coronavirus 2 (SARS-CoV2), was discovered in December 2019 in a person residing in the Chinese city of Wuhan. This person showed severe pneumonia symptoms. The World Health Organization (WHO) officially designated the illness as COVID-19 and labeled it a worldwide pandemic of significant concern [2].

The similarity between SARS-CoV-2 and coronaviruses found in bats and pangolins exceeds 90%, indicating a significant capacity for cross-species transmission [8]. The virus has a spherical morphology similar to other coronaviruses (CoVs) [9,10], measuring around 100 nm in diameter. It is classified as a single-stranded positive-sense RNA virus. It consists of four kinds of proteins: membrane (M) glycoprotein, spike (S) glycoprotein, envelope (E) glycoprotein, and nucleocapsid (N) protein [11]. Research revealed that the SARS-CoV-2 virus can attach to the angiotensin-converting enzyme 2 (ACE2) receptors. This binding process occurs in the presence of a protein called S protein and requires the involvement of the transmembrane protein serine protease 2 (TMPRSS2). Consequently, cells that express ACE2 receptors become vulnerable to viral infection [12,13].

It is essential to acknowledge that, besides impacting the airways, this newly developing virus can also have severe consequences for various other areas of the human body, such as the central nervous system (CNS). As a result, there is a potential escalation in susceptibility to neurological illnesses and neurodegenerative conditions [1,14]. The invasion of the CNS and its resulting pathology have been extensively investigated in infections caused by various viruses, including Japanese encephalitis virus (JEV), measles virus (MV), human immunodeficiency virus (HIV), and Varicella-Zoster virus (VZV), among others [15]. A growing interest exists in enhancing our understanding of the characteristics and mechanisms associated with neurological manifestations [16,17]. Patients with severe respiratory illnesses exhibit various clinical signs associated with neurological abnormalities, as outlined in Table 1.

Following the discovery of the first COVID-19 case in Wuhan, SARS-CoV-2 spread quickly worldwide and infected many people; according to WHO data, the global count of clinically confirmed COVID-19 cases exceeds 163 million, with a death toll of almost 3.3 million. Initially, COVID-19 was characterized as a respiratory disease presenting symptoms such as fever, tiredness, dyspnea, cough, and abnormal chest X-ray findings [18,19]. Additionally, many COVID-19 patients experience neurological symptoms such as ataxia, headaches, myalgia, drowsiness, hypogeusia, and hyposmia during infection [20,21,22]. SARS-CoV-2 has been extensively investigated as a causative agent for numerous neurological disorders [23].

However, the mechanisms by which SARS-CoV-2 impacts the CNS still need to be fully understood. This paper comprehensively analyses previously recorded CNS and PNS diseases associated with SARS-CoV-2. Additionally, we explore the various probable pathways of neuroinvasiveness, aiming to enhance neurologists’ understanding of the influence of SARS-CoV-2 on the CNS. This understanding will aid in diagnosing and developing appropriate treatment strategies for COVID-19.

**Table 1:** Neurologic manifestations associated with severe viral respiratory infections.

Respiratory Viruses	Virus Overview	Neurological Complications	Ref.
Human Coronaviruses (SARS-CoV1, MERS-CoV, SARS-CoV2)	➢ Coronaviridae family ➢ Positive-sense, single-stranded RNA viruses with an envelope	➢ Encephalopathy ➢ Encephalitis ➢ Meningitis ➢ Anosmia & dysgeusia ➢ Seizures ➢ Stroke ➢ Neuromuscular disorders	[24,25,26,27,28,29,30,31,32,33]
Influenza A Viruses (H1N1, H3N2, H5N1, H7N7)	➢ Orthomyxoviridae family ➢ Viruses with an enveloped negative-sense single-stranded RNA genome	➢ Encephalitis ➢ Seizures ➢ Guillain–Barré syndrome ➢ Reye’s syndrome ➢ Acute disseminated encephalomyelitis ➢ Encephalopathy ➢ Loss of consciousness ➢ Confusion ➢ Transverse myelitis	[34,35,36,37,38,39]
Human Respiratory Syncytial Virus/Orthopneumovirus	➢ Pneumoviridae family ➢ Virus with a filamentous enveloped, negative-sense, single-stranded RNA	➢ Encephalitis ➢ Seizures ➢ Status epilepticus ➢ Central apnea ➢ Encephalopathy	[37,40]
Human Metapneumovirus	➢ Pneumoviridae family ➢ A virus with a negative-sense, non-segmented, single-stranded RNA genome	➢ Encephalitis ➢ Focal seizures ➢ Status epilepticus	[41,42,43]

No matter what manifestations or damage in the CNS, neurologically impaired functions need to be restored. Clinical reports of novel neurorestorative treatments have recently improved the neurological processes of patients with CNS diseases and damage [44,45,46,47,48].

## 2. Respiratory Virus Transmission Routes to the Nervous System

The CNS serves as the primary regulatory entity of the human body and, therefore, requires specific measures to safeguard it against internal and external threats [49,50]. Hence, despite being closely related to the surrounding environment, the CNS is predominantly shielded from unrestricted infiltration of harmful substances, infections, and circulating antibodies in the bloodstream. This safeguarding is accomplished through the presence of the blood–brain barrier (BBB) and the blood–cerebrospinal fluid barrier (BCSFB), which are situated in the choroid plexus located within the brain’s ventricles [51]. BBB maintenance is ensured through tight junctions connecting the endothelial cells of cerebral microvessels, the astrocytic end feet, pericytes, and the extracellular matrix [51]. However, it has been noted that specific respiratory viruses possessing neurotropic characteristics can disturb the tight junctions of the BBB and ultimately invade the cells inside the CNS.

Moreover, it has been observed that these viruses can get past the BBB by directly infecting endothelial cells and pericytes through endocytic vesicles, a mechanism commonly referred to as transcytosis. An alternative method of accessing the CNS is the “Trojan horse approach”. This approach involves the exploitation of neurotropic viruses, which can cross the BBB by employing infected monocytes or macrophages, commonly referred to as hematogenous routes [52,53]. The section below will examine SARS-CoV-2’s various transmission routes and their mechanisms.

## 3. Coronavirus

The Coronaviridae family comprises a group of RNA viruses initially identified in the 1960s from individuals exhibiting symptoms of upper respiratory tract infections [54]. Human variants of coronavirus (HCoV), specifically alphacoronavirus and betacoronavirus, are prevalent globally and typically manifest with the characteristic symptoms of HCoV, such as rhinitis, pharyngitis, laryngitis, bronchitis, and otitis [55]. In 2002, a novel strain of coronavirus known as Severe Acute Respiratory Syndrome Coronavirus (SARS-CoV) was discovered. This particular strain was found to have originated from bats. It was transmitted to humans through an intermediate reservoir via zoonotic transmission [56], and in contrast to the usual Human Coronavirus (HCoV) strains, SARS-CoV exhibited an exceptionally high level of virulence. This was further exacerbated by acute respiratory distress syndrome (ARDS), multiple organ dysfunction syndrome, and a mortality rate reaching up to 10% in affected individuals [56,57]. Due to zoonotic transmission, the Middle East Respiratory Syndrome Coronavirus (MERS-CoV), a new coronavirus, was discovered in 2012. A 35% fatality rate has been noted for this specific coronavirus strain, which is linked to the emergence of severe respiratory symptoms [58]. In 2019, a novel coronavirus strain, SARS-CoV-2, was identified. The viral pathogen has undergone rapid dissemination, resulting in the emergence of a pandemic outbreak at the beginning of 2020. The virus exhibits a significant degree of homology with the SARS-CoV and is responsible for developing severe and often fatal pneumonia known as COVID-19. The clinical manifestations of this disease closely resemble those observed in cases of SARS-CoV and MERS-CoV [59].

### 3.1. How SARS-CoV-2 Infections Affect the CNS

#### The Neuronal Pathway

**Figure 1:**
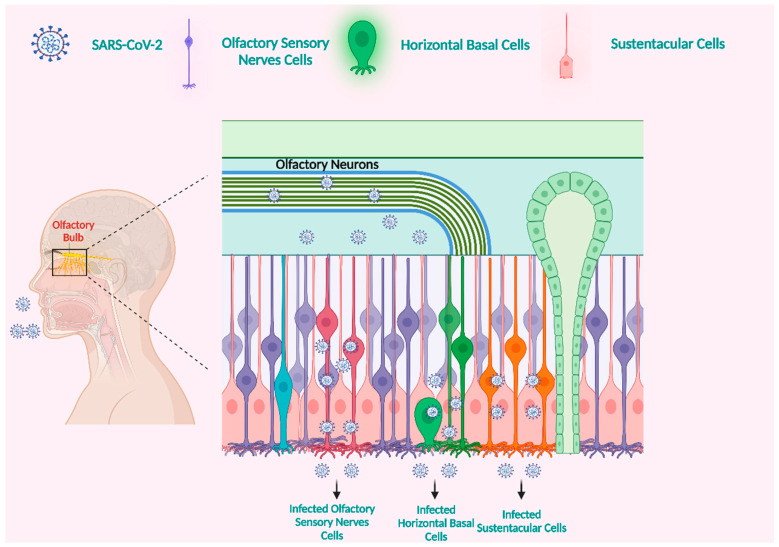
The olfactory nerve is a potential entry point for SARS-CoV-2 into the brain. The ACE2 receptor is involved in SARS-CoV-2 infection of the olfactory mucosa. Horizontal basal cells with the ACE2 receptor are everywhere across the olfactory epithelium. Infection with SARS-CoV-2 is likely due to the expression of ACE2 in human horizontal basal cells. Horizontal basal cells can develop into olfactory neurons. We propose that horizontal basal cells infected with SARS-CoV-2 can develop into infected olfactory neurons. These infected olfactory neurons have synaptic connections with olfactory bulb (OB) neurons. The central nervous system (CNS) could subsequently become infected. The OB is connected to numerous regions of the brain. This facilitates the spread of the virus to multiple brain regions quickly. Created with BioRender.com (https://app.biorender.com/illustrations/64901c58f8f3b377cd55200b, accessed on 28 August 2023).

The neuronal pathway serves as an essential pathway for neurotropic viruses to gain access to the central nervous system (CNS). Viruses have the ability to disseminate through the infection of sensory or motor nerve endings, employing retrograde or anterograde neural transport systems supported by motor proteins such as dynein and kinesins [53]. Olfactory neuron transport is an example of a neuronal pathway [2,60]. In the peripheral nervous system (PNS), the olfactory nerve is the primary pathway through which the SARS-CoV-2 virus infiltrates the CNS. This is primarily attributed to the higher presence of transmembrane protein serine protease 2 (TMPRSS2) and angiotensin-converting enzyme 2 (ACE2) within the olfactory epithelium cells. These proteins facilitate viral binding and accumulation [61,62,63,64]. The olfactory nerve can be classified as a CNS conduction loop rather than a typical nerve, as it establishes direct contact with the brain (Figure 1) [65,66]. The olfactory mucosa in the nasal cavity consists of neurons, basal cells, epithelial cilia, and Bowman’s glands [67,68]. The nasal cavity contains a unique neuroepithelium specialized for olfaction, characterized by sustentacular cells that primarily make up the apical surface [69]. Support cells were found to have high amounts of TMPRSS2 and ACE2 [70], demonstrating their susceptibility to SARS-CoV-2 infection [71].

In addition to the olfactory nerve, it has been suggested that SARS-CoV-2 may utilize other possible peripheral nerves, such as the nasopharyngeal nerves, trigeminal, and vagus, to access the brain. From an anatomical perspective, the vagus nerve is a component of the digestive nervous system and establishes connections with digestive pathways that exhibit heightened levels of NRP1 and ACE2 expression. ACE2 and TMPRRSS2 are found in intestinal enteric neurons and glia, suggesting their vulnerability to SARS-CoV-2 [72]. The gut–brain connection is significant in developing CNS diseases [73]. A comprehensive analysis of 42 individuals diagnosed with COVID-19 revealed that around 66.67% of the mentioned patients exhibited the presence of SARS-CoV-2 RNA in their stools [74]. In an experimental setting, it was demonstrated that SARS-CoV-2 can infect the epithelial cells of the human intestines [75]. Therefore, it is reasonable to conclude that enterocytes infected with SARS-CoV-2 may potentially disseminate to neuronal and glial cells within the enteric nervous system, ultimately leading to the invasion of the central nervous system via the vagus nerve [61,76].

### 3.2. Hematogenous Route

The hematogenous pathway is a potential path through which SARS-CoV-2 may gain access to the brain, as it involves circulating the virus within the bloodstream [1,2]. In this circumstance, the blood–brain barrier (BBB) is a frequently utilized pathway for disseminating the virus to the central nervous system (CNS). SARS-CoV-2 infiltrates the central nervous system (CNS) via the hematogenous pathway by employing two distinct mechanisms. Firstly, it infects vascular endothelial cells, allowing them to pass through the BBB. Secondly, it triggers inflammatory responses that lead to the disruption of the BBB.

#### 3.2.1. SARS-CoV-2 Infection of Vascular Endothelial Cells and Crossing the BBB

**Figure 2:**
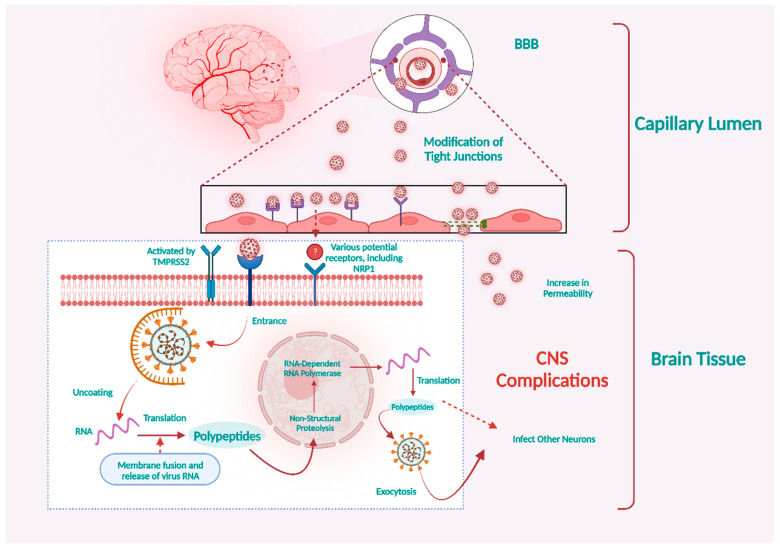
SARS-CoV-2 may infect vascular endothelial cells via the ACE2 or NRP1 receptors. Viral particles in the bloodstream can contaminate and replicate inside brain microvascular endothelial cells, allowing them to cross the BBB. Infection of neurons by SARS-CoV-2 and increased permeability of the BBB may account for COVID-19’s severe neurological manifestations. Created with BioRender.com (https://app.biorender.com/illustrations/64cb6825bac8184af357a74a, accessed on 28 August 2023).

Available reports show that the inflammation and disruption of epithelial barrier cells facilitate the virus’s ability to infiltrate the lymphatic vessels and circulatory system, subsequently leading to its dissemination to different organs, including the brain [23]. The autopsy of lung tissue from five patients who tested positive for COVID-19 showed the presence of viral proteins within the lungs’ capillaries. Furthermore, infection with the SARS-CoV-2 virus resulted in the death of endothelial cells and damage to the capillaries [77]. Although the incidence of SARS-CoV-2 detection in blood samples of COVID-19 patients remains relatively low, it does imply the potential for viral dissemination within the bloodstream and subsequent involvement of various organs, including the brain [78]. Upon entering the bloodstream, the virus can quickly infect the endothelial cells within the vasculature. This is facilitated by the presence of ACE2, TMPRSS2, and NRP1 expressions [79]. Additionally, a comprehensive examination of the postmortem analysis of a patient with COVID-19 revealed the presence of viral particles within both neural and capillary endothelial tissues of the frontal cortex. This indicates that the virus can enter the brain by infiltrating endothelial cells in the vascular system (Figure 2) [80]. Moreover, a laboratory study conducted using human blood vessel organelles revealed the invasion and replication of SARS-CoV-2. This finding supports the understanding of how infected brain endothelial cells facilitate the entry of blood-borne viruses into the brain [81]. The brain microvascular endothelial cells (BMVECs) represent a significant constituent of the BBB. The primary role of the BBB is to safeguard the brain by impeding the hematogenous infiltration of infections and toxic compounds into the CNS [82]. As a result, the virus must get past the BBB and subsequently infect the brain via the hematogenous pathway.

The choroid plexus demonstrated more excellent permeability in the blood–cerebrospinal fluid barrier than the BBB. Additionally, it was observed that the choroid plexus expressed ACE2 and TMPRSS2 [72], suggesting that it could serve as an alternative pathway for the invasion of the CNS by the virus.

Based on a study performed on a human choroid plexus model, it was observed that SARS-CoV-2 not only exhibited infectivity towards choroid plexus cells but also resulted in the disruption of the blood–cerebrospinal fluid barrier. This disruption presents an additional pathway for the virus to gain access to the brain [83].

#### 3.2.2. Immune Cells Initiate Cytokine Secretion in Response to SARS-CoV-2

**Figure 3:**
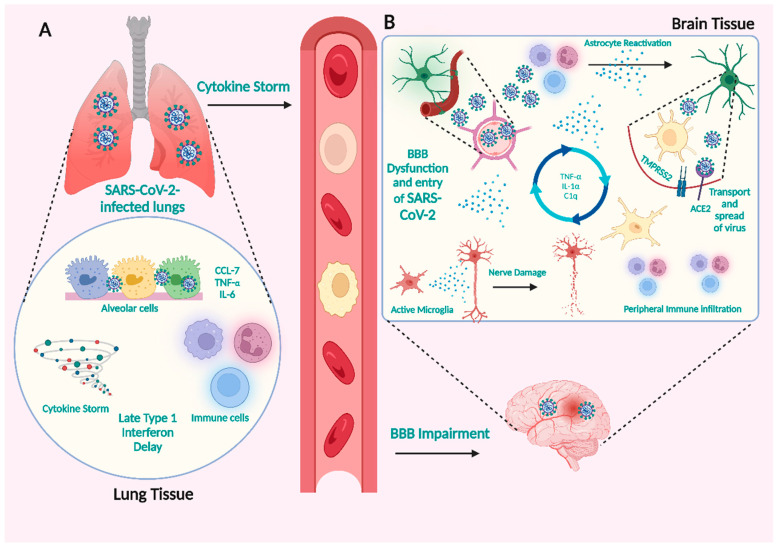
SARS-CoV-2 infection can result in increased peripheral immunological responses, which can lead to BBB disruption. (**A**) Cytokine storms with high BBB permeability may allow viruses or infected immune cells to enter the brain. (**B**) Potential CNS pathogenic processes generated by COVID-19-induced severe peripheral hyperinflammation. In COVID-19, infected immune cells penetrate the brain and produce cytokines that trigger glial cells, causing them to create pro-inflammatory cytokines, resulting in severe neurological symptoms. Created with BioRender.com (https://app.biorender.com/illustrations/64eb7c1e6661a54a2025c481, accessed on 28 August 2023).

The presence of a viral infection can lead to immune responses that have the potential to induce damage to the nervous system. It is noteworthy to mention that SARS-CoV-2 possesses the capability to infect immune cells, potentially leading to subsequent invasion of the CNS. The activation of different immune cells, such as neutrophils, macrophages/monocytes, T cells, and natural killer cells, has been observed in response to SARS-CoV-2. The immune cells that have been activated can eliminate the virus by releasing cytokines such as interferon (IFN), interleukin (IL), tumor necrosis factor (TNF), and chemokines [84,85,86,87,88]. Under typical biological conditions, it is observed that pro-inflammatory factors and immune cells can establish a positive feedback cycle, thereby contributing to the maintenance of cytokine balance [84,89]. However, it is essential to note that infection with SARS-CoV-2 has been observed to elicit an exaggerated immune response in specific individuals. This immune response can initiate a systemic inflammatory response characterized by cytokine storms (Figure 3). Consequently, the primary consequence of this inflammatory cascade is the infliction of damage to blood vessels [90,91]. Cytokine storms have distinct effects on BBB permeability, which may allow the virus or infected immune cells to reach the brain and induce associated CNS symptoms [92,93].

Previous studies have demonstrated that macrophages and peripheral lymphocytes, once infected, play a crucial role in facilitating the spread of the infection through the BBB, meninges, and choroid plexus [90,94]. According to reports, SARS-CoV-2 has predominantly infected human monocytes, while MERS-CoV has been observed to infect both T cells and monocytes. In the meantime, it has been established that SARS-CoV-2 can infect dendritic cells. Nevertheless, it was observed that both monocytes and macrophages exhibited a small level of ACE2 expression. This suggests an unidentified mechanism that potentially mediates the communication between the host’s natural immune system and SARS-CoV-2. The precise method through which SARS-CoV-2 contaminates immune cells is still not fully understood.

### 3.3. Expression of Essential Viral Infection Factors in the Nervous System

It is widely acknowledged that SARS-CoV-2 enters cells by utilizing ACE2 [12,21] with the involvement of TMPRSS2 [62,70], primarily due to the significant expression of these proteins in the pulmonary region. Moreover, extensive research suggests that the expression of both ACE2 and TMPRSS2 is also observed in the brain but at lower tiers. According to early immunohistochemistry research conducted by Lazartigue and colleagues, it was shown that ACE2 is present in the nerve cells of rat brains rather than the cells called glia [95]. Additionally, it has been determined that ACE2 plays a crucial role in regulating blood pressure and developing disorders related to the autonomic nervous system.

The investigation of human ACE2 mutant mice and brain organoids demonstrated that SARS-CoV-2 could infiltrate neurons and subsequently induce necrosis [96,97,98]. Furthermore, it is worth noting that the spike protein of SARS-CoV-2 can potentially engage with ACE2 receptors present in the endothelial cells of capillaries. This interaction raises the possibility that the virus could cause harm to the blood–brain barrier and gain access to the central nervous system by targeting the vascular system [99]. In addition to ACE2 and TMPRSS2, SARS-CoV-2 can invade other receptors or proteins, as shown in Table 2. Currently, the distribution of ACE2 is primarily determined by the analysis of mRNA data. However, it is essential to note that mRNA analysis does not fully capture the fully functional ACE2 protein distribution. Consequently, it is vitally necessary to conduct several immunohistochemical characterization studies.

## 4. Vascular Endothelial Growth Factor Cause Inflammation

**Figure 4:**
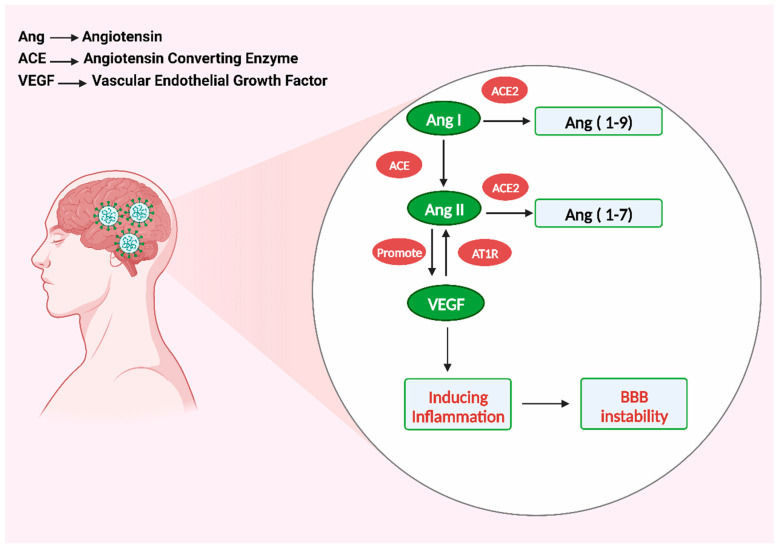
VEGF-induced inflammation damages the blood–brain barrier. Created with BioRender.com (https://app.biorender.com/illustrations/64e6045bf2e116b8a7af2194, accessed on 28 August 2023).

Vascular endothelial growth factor (VEGF) displays a widespread distribution within the CNS [115]. Its primary function is to regulate the processes of angiogenesis, proliferation of endothelial cells, and permeability of blood vessels [32]. Furthermore, the interaction between SARS-CoV-2 and ACE2 can activate the renin–angiotensin system, a pathway implicated in the inflammatory response (Figure 4). This activation subsequently facilitates the production of VEGF using the binding between angiotensin II (AngII) and angiotensin II type 1 receptor (AT1R). In reality, VEGF increases angiogenesis in brain disorders and damages the BBB by causing inflammatory reactions [116].

Angiogenesis is generally accompanied by inflammation, which causes an increase in vascular permeability and the recruitment of inflammatory cells [116]. The ACE2 enzyme plays a crucial role in the catalytic process of converting Ang I and Ang II, Ang I to Ang 1-9 and Ang II to Ang 1-7, respectively [117,118]. When the SARS-CoV-2 virus interacts with ACE2, it can deactivate this enzyme, which may cause an increase in the activation of the ACE/AngII/AT1R axis, subsequently leading to the excessive synthesis of AngII. The positive feedback of Ang II stimulated the growth of ACE2 in the brain infected with SARS-CoV-2. VEGF, in turn, boosts Ang II, resulting in a cycle that releases pro-inflammatory cytokines such as TNF-, IL-1, IL-6, IL-8, and ICAM-1 [119]. Furthermore, interleukin-6 (IL-6) is an important inflammatory cytokine mediator linked to the severity of COVID-19 symptoms. It can be used as an indicator of COVID-19 severity [87,120].

## 5. Neurologic Symptoms of SARS-CoV-2 Infection

**Figure 5:**
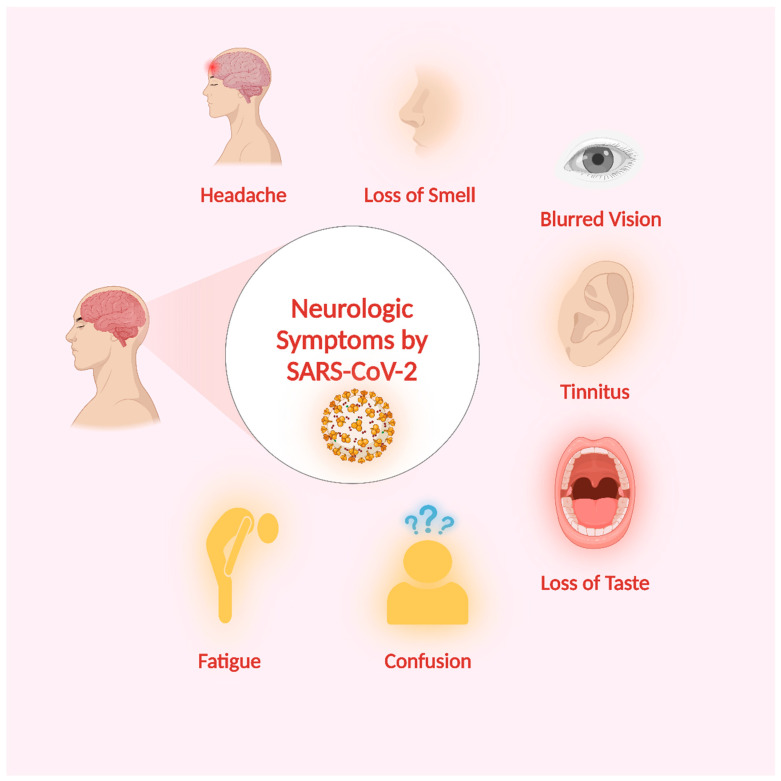
SARS-CoV-2-related neurological symptoms. Created with BioRender.com (https://app.biorender.com/illustrations/649345fe5442443eead4ecc9, accessed on 28 August 2023).

According to available data, the capability of SARS-CoV-2 to infect the neurological system is becoming more apparent. Case reports and retrospective cohort studies have been the primary sources of information regarding neurological symptoms of SARS-CoV-2 infection. China has conducted the first retrospective investigation on neurological symptoms [28,121,122]. Among the study group of 214 individuals diagnosed with COVID-19, it was observed that 78 patients, or 36.4% of the study, displayed neurological symptoms. The study revealed that individuals had acute cerebrovascular disease, impaired consciousness, skeletal muscle injury, and neurological symptoms, including dizziness, headache, nausea, blurred vision, tinnitus, fatigue, decreased taste sensation, and reduced sense of smell [123] (Figure 5). One of the symptoms of COVID-19 is headache, with a frequency of 6–13% [109,124,125,126,127]. It frequently occurs with other symptoms like fever and cough; thus, it is not an isolated symptom. Hyposmia and hypogeusia were noted as symptoms observed in individuals diagnosed with COVID-19, with reported prevalence rates varying from approximately 5% [28,128] to as high as 70% [129] or even exceeding 79% [130,131]. The data presented in this study indicate that specific findings may possess prognostic relevance in predicting the possibility of serious neurological problems. However, further research needs to be done in prospective studies to assess the diagnostic significance of neurological symptoms. This would greatly facilitate the early identification of patients at risk for neurological complications.

## 6. SARS-CoV-2-Related Disorders of the Nervous System

**Figure 6:**
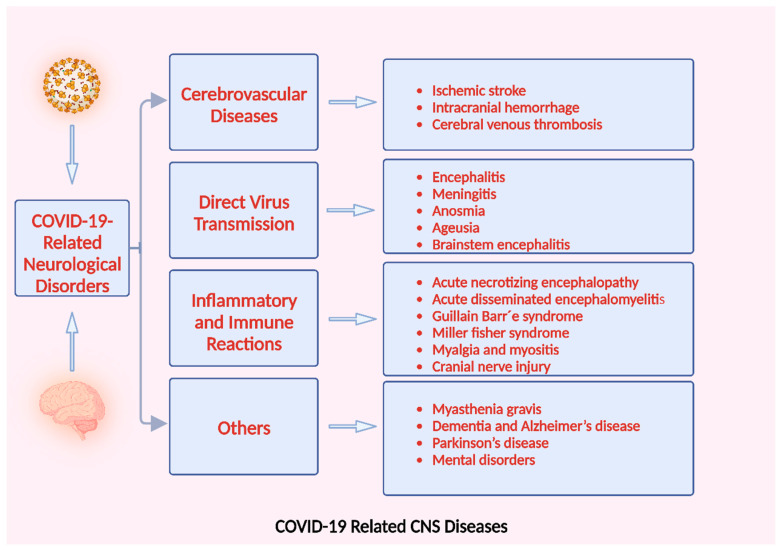
CNS diseases associated with COVID-19. Created with BioRender.com (https://app.biorender.com/illustrations/6494a0a2b6f253735e3e3b2a, accessed on 28 August 2023).

The possible neurological diseases induced by SARS-CoV-2 fall into three broad categories: (a) the neurological adverse effects of associated pulmonary and systemic disorders, such as cerebrovascular disease; (b) the virus directly infiltrates the CNS, resulting in encephalitis; and (c) possible immune-mediated conditions following an infection, like Guillain–Barre syndrome (GBS) and other demyelinating diseases (Figure 6).

### 6.1. Cerebrovascular Disease

**Figure 7:**
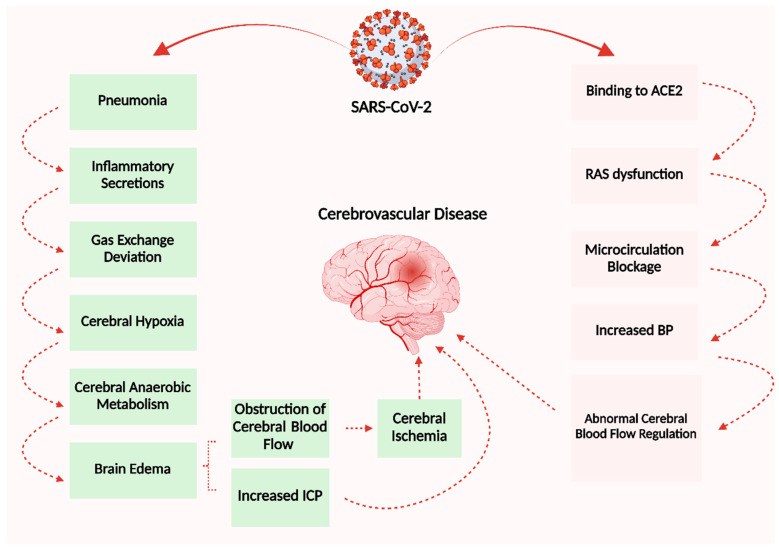
This schematic diagram illustrates the pathophysiological mechanisms underlying cerebrovascular disease resulting from SARS-CoV-2. Created with BioRender.com (https://app.biorender.com/illustrations/64e779e77fc48876a30e4ac2, accessed on 25 August 2023).

Cerebrovascular disease is a group of diseases that affect the vessels that carry blood to the brain and induce brain tissue injury due to disruptions in cerebral blood circulation [132]. The virus replication within the pulmonary tissue induces extensive alveolar and interstitial inflammatory fluids and the production of hyaline membranes. This will result in abnormal alveolar exchanges of gases, hypoxia of the CNS, an increase in the anaerobic breakdown of brain tissue, the onset of edema between cells, blockage of cerebral circulation, resulting in ischemia of cerebral circulation, and gradual deterioration of brain function as the pressure in the brain rises [120,133,134,135]. It can also cause acute cerebrovascular disorders like cerebral venous thrombosis, hemorrhage, and ischemic stroke (Figure 7).

**Figure 8:**
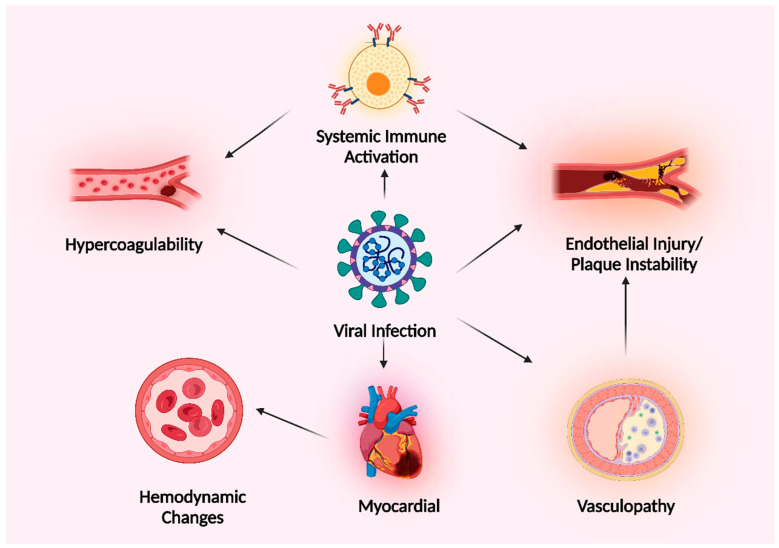
A diagram demonstrating the different methods through which viral infections might cause ischemic stroke. Created with BioRender.com (https://app.biorender.com/illustrations/64cd3a53449ef0d0fd42f67d, accessed on 28 August 2023).

Ischemic stroke is associated with many different viruses [136]. Stroke risk is increased even though the infection is often localized to the periphery and there is no evidence of the virus in the CNS. In these situations, systemic immunological activation is assumed to be the primary pathogenic mechanism. Combined with the accompanying hypercoagulability or endothelial dysfunction, this may result in vascular damage or the development of thromboemboli [137,138]. In such cases, the pathogenic mechanism of stroke may be easier to explain; however, CNS invasion does not rule out a role for systemic immune activation in stroke pathogenesis (Figure 8).

Acute ischemic stroke (AIS) is an emergent vascular complication in COVID-19 patients, with reported incidence rates ranging from 1% to 6% among hospitalized patients [139]. COVID-19 patients are more likely to have a stroke and have more severe symptoms and outcomes [140]. The classification of COVID-19 with ischemic stroke is based on two distinct classes determined by the underlying mechanism of occurrence.

The first category comprises senior individuals with a medical background with numerous coronary and cerebrovascular complications or who exhibit significant comorbidities before contracting the virus. These individuals are more susceptible to pulmonary embolism incidents [141]. The second category primarily comprises relatively young individuals without coronary and cerebrovascular factors or associated medical conditions before contracting the SARS-CoV-2 virus. This might be connected to how SARS-CoV-2 affects various bodily systems [142,143,144]. The coagulation process is essential in this phenomenon since around 25% of cases show evidence of systemic coagulation [145].

The initial inquiry explored the neurological signs observed in patients diagnosed with COVID-19 in Wuhan, China, which was the pandemic’s epicenter. The study revealed that out of the 214 patients included in the analysis, 78 individuals (36.4%) experienced neurological problems [28]. Patients with severe COVID-19 exhibited a higher prevalence of acute cerebrovascular disease than individuals with non-severe manifestations. It is important to note that individuals with severe illness showed higher D-dimer levels than non-severe infection [28].

The incidence of hemorrhagic stroke in individuals with COVID-19 is lower when compared to ischemic stroke. However, the causal link between COVID-19 infection and hemorrhagic stroke has not been demonstrated. Researchers performed a retrospective analysis of 11 individuals with acute cerebrovascular disease who had contracted COVID-19 in Wuhan. Among the patients, a 60-year-old man was identified as having a brain hemorrhage 10 days after a severe COVID-19 infection. The patient died 13 days after the stroke because of a high BP level (150/80 mmHg) [146].

According to Sharifi-Razavi et al., a 79-year-old man with a fever, cough, and acute loss of consciousness 3 days later was recorded. Without a history of elevated blood pressure or taking anticoagulant medication, the patient was admitted with a BP of 140/65 mmHg. In addition to intraventricular and subarachnoid hemorrhages, a CT scan revealed a severe intracerebral hemorrhage (ICH) in the right ventricle. However, CSF analysis was not performed despite the oropharyngeal swab revealing COVID-19 infection [147].

Cerebral venous thrombosis (CVT) is less common than cerebral infarction and hemorrhage [148,149], with a total frequency of 0.3% [150]. COVID-19 causes a hypercoagulable condition and systemic thrombosis, which includes CVT. Under the influence of COVID-19, patients without congenital CVT risk factors can develop excessive coagulation and thrombosis, leading to CVT [151,152]. These results collectively suggest that COVID-19 can cause cerebrovascular episodes, even though further thorough research is urgently needed. It is crucial to understand this information to avoid and treat the symptoms of cerebrovascular illness in COVID-19 individuals.

### 6.2. CNS Diseases by Direct Virus Transmission

Encephalitis is defined as inflammation of the brain parenchyma resulting from infections, which include neuronal destruction and nerve cell injury. The observation of viral encephalitis in many individuals who have contracted SARS-CoV-2 has led to speculation over the potential existence of this neurological consequence [153,154,155,156,157]. The presence of SARS-CoV-2 within the CSF of patients infected with COVID-19 was confirmed by the medical team working at Beijing Ditan Hospital using genome sequencing. This confirmation provided clinical evidence of viral encephalitis [120]. This makes it very likely that SARS-CoV-2 will cause encephalitis. However, images of brain tissue from SARS-CoV-2 cases showed no signs of inflammation [158].

Several autopsy findings indicate the presence of lymphocytic panencephalitis, meningitis [159], partial neuronal atrophy, and brain edema [160]. Around cerebral blood arteries, sparse or significant clumps of inflamed cells, mainly monocytes, have been seen. Soft focal meningitis is also found [161].

The prevalence of anosmia and ageusia is significantly elevated among people diagnosed with COVID-19. The prevalence of anosmia is more than 85.6%, whereas that of ageusia is reported to be 88.0% [130]. Some research studies believe this phenomenon can be attributed to the degeneration of olfactory sensory neurons resulting from many factors, such as dysfunction of supporting cells, apoptosis triggered by inflammation, or potentially direct infection [162]. However, the mechanism remains unclear. Symptoms in individuals with mild COVID-19 lasted approximately 10 days, with 89% of patients recovering within four weeks after diagnosis [163].

Brainstem encephalitis (BE) is a rare, severe, and quickly spreading inflammatory condition affecting the brainstem. Multiple experiments and animal models have indicated the potential transmission of SARS-CoV-2 to the brainstem nucleus via various pathways, including the olfactory nerve, trigeminal nerve, facial nerve, glossopharyngeal nerve, vagus nerve, dorsal root ganglia, etc. However, the evidence remains insufficient to support this hypothesis conclusively [141]. Brain autopsy findings revealed the presence of neuronal cell death and axonal degeneration exclusively inside the cerebral cortex. Further research is required to prove the association between COVID-19 and encephalitis, as the current understanding is based on theoretical pathways proposed in clinical observations and neuronal colonization.

### 6.3. Nervous System Damage Induced by Abnormal Immune and Inflammatory Reactions

The etiology of numerous neurological disorders can be linked to increased inflammation and immunological dysregulation resulting from infection. COVID-19 is not an exception. Acute necrotizing encephalopathy (ANE) is an uncommon disease that typically develops after a critical febrile illness, most commonly a viral infection, and is characterized by brain damage (encephalopathy) [164]. The predominant imaging characteristics widely observed include the presence of symmetrical multifocal lesions and the engagement of the thalamus [29]. Autopsy findings indicate the presence of significant vasculitis, characterized by varying levels of segmental and complete destruction of the endothelial cells. Thrombosis primarily affects the vascular bed’s microcirculation. Additionally, there is significant evidence of hemorrhagic necrosis, inflammation, and severe necrotizing damage to the neurons. Localized cerebral edema may also be present [165].

Guillain–Barré syndrome (GBS) is an uncommon autoimmune condition characterized by acquired nerve damage caused by the immune system, resulting in muscle weakness and, in some cases, paralysis. In COVID-19 patients, most GBS variants have been identified [166]. According to specific research, 60% of GBS patients achieve partial to full recovery [167]. In addition to its connection to ACE2, SARS-CoV-2 has been observed to require sialic-acid-containing glycoproteins and gangliosides for cellular entry [168]. The pathophysiology of GBS is tightly associated with these two sites as well. Hence, it is possible that cross-reactivity could manifest as a viable method by which SARS-CoV-2 may induce GBS [168].

Myalgia is a frequently encountered symptom seen in individuals diagnosed with COVID-19. The condition’s prevalence varies from 3.36% to 64%, with an estimated total prevalence of 19.3% [150,169]. In rare instances, myalgia and muscle injury have the potential to advance into the condition known as rhabdomyolysis [170]. The expression of ACE on skeletal muscle may be the primary cause of myalgia [171]. Further diagnostic investigations, including muscle biopsy and antibody screening, are required for individuals diagnosed with COVID-19 who exhibit indications of skeletal muscle damage.

### 6.4. Other COVID-19-Related Neurological Disorders

Myasthenia gravis (MG) is a persistent autoimmune condition characterized by the destruction of nerve–muscle communication by antibodies, leading to skeletal muscle weakening. Two new MG cases were identified in a survey of approximately 11,000 COVID-19 patients [172]. The mechanism may be connected to immunological dysfunction and infection-induced inflammation [173]. In addition, the research findings of another study revealed that out of 3558 patients diagnosed with Myasthenia Gravis (MG), 34 patients contracted the COVID-19 virus at a low rate (0.96%) [174]. Most MG patients become more seriously ill after contracting COVID-19. A total of 73% required mechanical ventilation, 87% required ICU care, and 30% passed away [175].

Multiple Sclerosis (MS) instances among individuals diagnosed with COVID-19 are rare. In a recent study, only two cases were documented out of a sample size of approximately 11,000 individuals [172]. In the context of multiple sclerosis (MS), it is essential to examine the potential impact of disease-modifying therapy (DMT) on the vulnerability of MS patients to contracting COVID-19. Nevertheless, it is necessary to note that the available data on (MS) are limited in scope, and further research is needed.

According to recent studies, the prevalence of dementia among individuals diagnosed with COVID-19 is reported to be 0.67% [176]. The average rate of newly diagnosed dementia within 14–90 days after COVID-19 among individuals aged 65 and above is reported to be 1.6% [177]. There is a significant correlation between dementia and the COVID-19 pandemic. Specific signs and symptoms associated with Alzheimer’s disease and related dementia (ADRD) can heighten susceptibility to COVID-19 infection. These symptoms encompass an impaired capacity to understand and comply with COVID-19 prevention instructions and standards and alterations in personality and memory [178,179].

## 7. Conclusions and Future Perspectives

COVID-19 presents a significant challenge to the global community. The investigation of the neurological implications of SARS-CoV-2 infections is a rapidly growing field among neuroscientists. Even though SARS-CoV-2 is primarily responsible for respiratory problems, mounting evidence points to the possibility of a neuroinvasion by this virus. SARS-CoV-2 isolation from CSF, the discovery of the virus on olfactory receptors, and numerous neurologic manifestations in COVID-19 patients have all provided evidence. CNS and PNS problems have reportedly been noticed in these patients. However, it is difficult to determine if CNS symptoms were caused by CNS infection or peripheral infection because of blood clotting, hypoxia, and cytokine storms in severe patients. Furthermore, the method by which SARS-CoV-2 invades the nervous system still needs to be better understood.

Currently, the potential pathways of SARS-CoV-2 neuroinvasion are:

✔ Entry via the olfactory nerve;
✔ Direct infection of vascular endothelial cells;
✔ The initiation of inflammatory reactions that breach the BBB facilitates invasion.

All of the above routes are linked to ACE2 or NRP1; hence, the best optimal strategy for investigating the mechanism by which SARS-CoV-2 infiltrates the CNS would involve determining the respective distributions of NRP1 and ACE2. Understanding this wealth of knowledge is essential for preventing and controlling CNS symptoms and facilitating the rehabilitation process for those affected by COVID-19.

It is evident that the knowledge we have acquired thus far only scratches the surface, and there is still a significant amount of information to be comprehended in this field to facilitate effective therapeutic intervention.

## Figures and Tables

**Table 2:** SARS-CoV-2 infection-related receptors or proteins in the CNS.

Receptors/Protein	Primary Expansion Area	Reference
ACE2	Hypothalamus, Pituitary Gland	[100,101,102,103,104,105]
TMPRSS2	Cerebellum, Hypothalamus, Pituitary Gland	[70,106,107]
NRP1	Olfactory Bulb	[108]
BASIGIN	Pituitary Gland, Frontal Cortex	[109,110,111,112]
Cathepsin L	Pituitary Gland, Spinal Cord	[113,114]

## Data Availability

Not applicable.
